# Happy birthday, Acta!

**DOI:** 10.3109/17453671003685392

**Published:** 2010-03-31

**Authors:** Anders Rydholm, Olle Svensson

**Affiliations:** anders.rydholm@med.lu.seolle.svensson@orthop.umu.se

## Perspective

80 years ago, Acta Orthopaedica Scandinavica was founded by an enlightened group of orthopedists who thought that Scandinavia also needed an orthopedics journal. Already from the beginning, they understood that orthopedics and science were international matters, and for many years Acta articles were therefore published in either English, French, or German, with abstracts in all languages. This international view was merely confirmed a couple of years ago, when we decided to drop “Scandinavica”, since for a long time most of the manuscripts had been coming from countries outside Scandinavia. The name change to Acta Orthopaedica also marked that the Netherlands and Estonia had joined the Nordic Orthopaedic Federation (originally an alliance between the national orthopedic societies of Denmark, Finland, Iceland, Norway and Sweden), which owns Acta Orthopaedica, a non-profit journal. Our ownership gives us a certain amount of freedom to make our own decisions. When the Acta board a couple of years ago decided that we should go free on the internet, without any cost to authors, our esteemed publisher (Informa Healthcare) became worried; the poor house was around the corner! When we as the owners insisted, the publisher said: “OK, do that, but just as long as you don't tell anybody!” However, knowledge is like manure—it doesn't do any good if it is not spread widely. Acta articles are now searchable in PubMed as soon as the proofs are ready. Of course, this reduces the number of subscriptions. So far, we have managed to make ends meet, but some time in the future we may have to rely on a small page charge. Anyway, it will be a reasonable cost considering what many commercial journals charge authors today.

We still believe that paper journals will outlive their readers—at least the elderly ones. We still print a paper edition; the bulk of these books are sent to the members of the Federation and to a number of libraries. But the internet is the stage for science. Searchability and swiftness make up for the nice-looking leather-backed volumes that have been around since 1930. Today, you have the entire Acta production since the very start in 1930 at your fingertips (http://informahealthcare.com/ort). If they had been able to log in, the founding fathers (there were few orthopedic mothers at that time) would probably have been more than a little bit impressed by the new technology and the clinical results, results that they would never ever have dared to dream of. At the same time, they would perhaps think that part of what we are doing today seems a little bit off-off Broadway, so to speak, considering the disease horizon of their time. The evolution of clinical articles is illustrated in this issue (pp 15–20) by a review on the treatment of the displaced femoral neck fracture, as reflected by Acta articles published 1932–2008. Our articles range from case reports to randomized clinical studies and national, or even multinational, register studies.

What is really important, though, is what future readers, bloggers, twitters, hackers, crackers, or whatever, will think. There is only one thing one can be sure of, and that is that it will be different. The great physicists and physicians at the end of the nineteenth century were convinced that everything worth knowing was already known then; there were only some details that might need some minor adjustment. At Acta's centennial the maps have been redrawn, hopefully with new white uncharted areas. What about the future? You decide. All communication takes place according to the conditions of the receiver—you.

## Acta specialties

### Open Access

As previously mentioned, Acta Orthopaedica is owned by the Nordic Orthopaedic Federation and is a non-profit journal with immediate Open Access. It is not only cost-free to the general reader but also to authors. Likewise, something that is also free to authors is that we print in color and we actually require figures in color (which, 25 years ago, came as a surprise to pathologists in particular who were used to seeing colors under the microscope all day but printed matter in black and white).

### Creative commons

Acta articles are distributed under the terms of the Creative Commons Attribution Non-commercial License, which permits any non-commercial use, distribution, and reproduction in any medium, provided the source is credited. This means that Acta has no copyright on your article; you as an author, and others, are free to use it for all non-commercial purposes.

### Statistics

Almost all clinical and laboratory experiments require statistical considerations for planning and evaluation. It has been shown repeatedly that articles—even in high-ranking journals—surprisingly often contain inadequate statistics. Since 1993, Acta has been taking advantage of an experienced biostatistician (Jonas Ranstam; see also page x-y in this issue) to make sure that the conclusions presented in a manuscript are supported by the data, and that any weaknesses and limitations imposed by the study design, data collection, and statistical analysis are pointed out to the reader.

We require transparent and accurate reporting of research studies, and recommend that our authors should comply with the reporting guidelines CONSORT (for clinical trials), STROBE (for observational research), STARD (for diagnostic accuracy studies), and PRISMA (for systematic reviews). We do not like presentation of results with p-values as the only characterization of findings, and prefer presentation of estimated effects with 95% confidence intervals. We dislike the ambiguous use of terms that do not appear to have a clear definition, such as “significant” (does this indicate a statistically significant difference, a clinically important difference, or both?) and “statistical difference” (all reported differences, statistically significant or not, are in some sense statistical). After all, a difference is a difference that makes a difference.

### Registry studies

In this issue, we present 20 registry studies from all over the world, mainly on arthroplasties. The first national arthroplasty registry studies were implemented in Sweden: the Knee Register in 1975 (on the iniative of Göran Bauer, former editor of Acta), followed by the Hip Register in 1976. The history of these registries is presented on pp 3–7. Göran Walldius in Stockholm was one of the pioneers of knee arthroplasty; one of his articles is reprinted in this issue as a “classic” (pp 21–33).

The strength of registry data as compared to other types of clinical studies is discussed in a Guest Editorial (pp 8–9) and the appropriate use of statistics in these studies is discussed on pp 10–14. We firmly believe that Acta, with its Open Access, is an appropriate journal for effective dissemination of these registry studies!

